# A negative association between prevalence of diabetes and urban residential area greenness detected in nationwide assessment of urban Bangladesh

**DOI:** 10.1038/s41598-021-98585-6

**Published:** 2021-09-30

**Authors:** Jahidur Rahman Khan, Amena Sultana, Md. Mazharul Islam, Raaj Kishore Biswas

**Affiliations:** 1 Biomedical Research Foundation, Dhaka, Bangladesh; 2 Institute of Statistical Research and Training, University of Dhaka, Dhaka, Bangladesh; 3 Bangladesh Institute of Governance and Management, Dhaka, Bangladesh; 4 Transport and Road Safety (TARS) Research Centre, School of Aviation, University of New South Wales, Kensington, Australia

**Keywords:** Diseases, Risk factors, Environmental impact

## Abstract

Residential area greenness may influence diabetes, but limited studies have explored this relationship in developing countries. This study assessed the association between residential area greenness and diabetes among urban adults in Bangladesh. The mediation effect of the body mass index (BMI) was also assessed. A total of 2367 adults aged ≥ 35 years were extracted from a nationally representative survey. Diabetes was characterised as fasting plasma glucose level be ≥ 7.0 mmol/L or taking prescribed medications to reduce blood sugar level. Residential area greenness was estimated by enhanced vegetation index. Binary logistic regression models were employed to estimate the association between residential area greenness and diabetes adjusting for sociodemographic factors. Mediation analysis was performed to assess whether BMI mediated the association between greenness and diabetes. Greater area greenness was associated with lower odds of diabetes (adjusted odds ratio 0.805, 95% confidence interval 0.693–0.935, p = 0.0052). BMI significantly mediated 36.4% of the estimated association between greenness and diabetes. Presence of areas of greenness adjacent to living area tends to be associated with lower diabetes prevalence. Findings emphasised the importance of preserving the local environment to tackle the growing diabetes prevalence in Bangladesh.

## Introduction

Population projections suggest that by 2050, there would be about 7.4 billion people living in urban areas globally^1^. Bangladesh had an estimated urban population of 53 million in 2014, projected to double by 2050 with an estimate of 112 million^1^. This rapid urbanisation is expected to magnify the prevalence of noncommunicable diseases (NCDs), including diabetes and risks associated with NCDs. Since urban areas are becoming home to larger populations, the influence of built environment on NCD, particularly diabetes among urban dwellers in Bangladesh, requires a much needed assessment.

Bangladesh has experienced a substantial shift in diabetes prevalence. According to the national survey 2011 Bangladesh Demographic and health survey (BDHS), the diabetes prevalence among adults aged 35 + years was 11% (11.2% women and 10.7% men)^2,3^. These figures translated to 5 million people with diabetes which is projected to be 8 million by 2025^3^. The latest 2019 International Diabetes Federation Diabetes Atlas reports that already 8.1% of adults aged 20–79 years had diabetes in Bangladesh. Also, Bangladesh ranks among the top 10 countries for diabetes cases among 20–79 years aged (approximately 8.4 million) and is projected to be nearly double by 2045 (approximately 15.0 million)^4,5^.

Besides premature morbidity and death, diabetes places an enormous burden on health care systems. Diabetes is a major cause of stroke, renal failure, visual impairment, and neuropathy^6^. In 2019, about 109,857 estimated deaths were attributable to diabetes and related complications among adults (aged 20–79 years) in Bangladesh^5,7^. Between November 2016 and October 2017, diabetes-related hospital admissions rate was 3.75 (per 100,000) in Bangladesh^4^. Besides, the average diabetes-related health expenditure per person was US$ 63.9, and the total diabetes-related health expenditure was US$ 535 million in 2019^7^.

Patients with diabetes had more days of inpatient treatment, more hospital visits and more medications than patients without diabetes in Bangladesh^8^. The estimated annual per capita spending on medical treatment for patients with diabetes (US$ 635) was 6.1 times higher than patients without diabetes (US$ 104)^8^. Diabetes prevalence in urban areas is significantly higher than in rural areas, which could be due to sedentary lifestyles, physical activities, dietary habits (overconsumption of fast and fatty food) and rising adiposity^9^.

With a higher prevalence of diabetes among urban adults, preventive approaches based on a better understanding of the aetiology of diabetes might be more cost-effective with greater gains than treatment. The aetiology of diabetes is multidimensional; previous studies have identified several genetic, environmental, behavioural, sociodemographic, anthropometric and dietary factors associated with diabetes^10–15^. In addition to interventions on individuals promoting healthy lifestyles, enhancing built-environmental features of the residential area can reduce diabetes^16^.

Built-environmental features of the residential area, such as green space or greenness, could impact the prevalence of diabetes, although the mechanism is yet to be fully developed. Earlier epidemiological studies have shown that greater green space or greenness has protective effects against diabetes^17–23^. Associations between green space or greenness and diabetes could be attributable to lower air pollution, enhanced physical activity and reduce adiposity^24^. Air pollution, sedentary lifestyle, lack of exercise, overweight, and excessive caloric diet consumption are associated with diabetes^10–12,15,25–27^.

The availability of wide green spaces is becoming popular as a preventive measure for diabetes. The main hypothesis is that presence of green areas will lead to fresher air, access to fresh, green vegetables and fruits, as well as invite more active outdoor exercises^20,28–30^. Previous studies found that adults living near green spaces and parks are more active and maintained healthy body weight because of regular physical exercise^31,32^. In this backdrop, urban community greenness may be viewed as an important factor against diabetes.

Studies that explored the nexus between green space and diabetes are conducted in developed countries^17–20,23^. Despite the growing literature on the effects of green space or greenness, evidence from developing countries remains limited. Studies in China reported that higher residential greenness is associated with a lower diabetes prevalence among adults^21,22^. A South Indian study reported that greater built-up land use (or reducing greenspace) surrounding residences were associated with higher fasting glucose among adults, although the association was not statistically significant^33^. However, there is a dearth of similar studies on Bangladesh. A study of adults sampled across three cities—Sydney, Singapore, and Dhaka—found that more sedentary activity and walking was associated with higher quality green space^34^. A negative association was reported between the objectively measured vegetation coverage and the mental wellbeing among adults in in nine slums of Dhaka, Bangladesh^35^. The intensity of greenspace use was reported to be higher among people with noncommunicable diseases, and park prescriptions mediated this relationship among middle-aged people in Dhaka^36^. These studies have been limited to Dhaka, Bangladesh's capital, and have not evaluated the relationship between greenness and diabetes exclusively.

Since Bangladesh is experiencing rapid urbanisation and rural to urban migration, and green infrastructures (e.g., parks, water bodies) are also quickly converting to grey infrastructure (e.g., buildings). In many cases, green spaces such as parks, playgrounds and waterfronts have been invaded, occupied or capsized by poor homeless people to create slums or by business owners and political leaders. Therefore, urban residents are losing opportunities to interact with green infrastructure that promotes health and well-being^37^.

The unplanned development of roads without footpath, trees and green space has hampered walkability and physical activity opportunities in Bangladesh’s metropolitan cities, namely Dhaka and Chittagong. The research revealed a high prevalence of insufficient physical activity among the adult population of Bangladesh, and urban residents are more likely to have inadequate physical activity than those living in rural areas^38,39^. Moreover, vehicular traffic in urban areas has risen exponentially, resulting in an increased level of emissions-related air pollution, leading to adverse effects on urban residents.

Bangladesh government has taken a multisectoral action plan for NCD control and prevention, where the key target is to address the four main NCD diseases, including diabetes^40^. To achieve these targets, assessing the potentially protective effect of greenspace (or greenness) on diabetes in urban Bangladesh could be beneficial.

This study aimed to assess the association between residential area greenness and diabetes among urban adults in Bangladesh. Also, this study assessed whether the relationship between greenness and diabetes was mediated by the body mass index (BMI). Findings from this study will help advise policymakers to consider greenspace management as part of diabetes prevention policy.

## Methods

### Data

To assess the relationship between greenness and diabetes, this study was drawn on data from the Bangladesh Demographic and Health Survey (BDHS) of 2011, which aimed to collect nationally representative data on various health, nutrition, lifestyle and sociodemographic indicators. A two-stage stratified cluster sampling was used to draw the sample, where enumeration area (EA) or survey cluster were selected based on the most recent census EAs prepared by the Bangladesh Bureau of Statistics^2^. In the first stage, 600 EAs (207 urban and 393 rural) were selected with the probability of selection proportional to the EA size, and on average, 30 households per cluster were selected by systematic random sampling in the second stage. A total of 17,141 households were successfully interviewed^2^. One in three of the eligible households was selected for biomarker measurement (e.g. blood glucose). A subsample of 8835 females and males aged 35 years and older were eligible to participate in the biomarker components. Details of the sampling strategy and biomarker measurement are available in survey report^2^. In addition, environmental features were collected at the survey cluster level. As an indicator of residential area greenness, we considered survey cluster-level enhanced vegetation index (EVI) for the year 2010^41^. This study focused on urban areas; therefore, a total of 2367 participants were selected from urban regions after applying exclusion criteria. Exclusion criteria included non-usual residents of households, non-pregnant, no information about blood glucose, diabetes medication, BMI, living in a residential area without having an EVI value, and missing values in covariates.

This study has been used the secondary data set of the 2011 BDHS, which was conducted through a joint collaboration. Ethical approval of conducting this survey was taken from the ICF International Institutional Review Board and Bangladesh Medical Review Council. The authors had taken permission for and the data set is publicly available in DHS program website (https://dhsprogram.com/Data). In addition, spatial data are freely available in spatial data repository of the DHS program (https://spatialdata.dhsprogram.com/covariates/).

### Measures

#### Diabetes

The outcome variable of interest was ‘diabetes’. An individual was classified to have diabetes if his/her fasting plasma glucose value was ≥ 7.0 (mmol/L) or currently taking diabetes medication. In 2011 BDHS, HemoCue 201 + blood glucose analyser was used to measure adults’ blood glucose levels. Detail description of collecting a blood sample is available in survey report^2^.

#### Greenness

Residential area greenness was defined using survey cluster level enhanced vegetation index (EVI), which was extracted based on the raster extraction method by the DHS authority^41^. A circular buffer with a 2-km radius was drawn around urban cluster location points. All raster cells whose centroids fall within the buffer surrounding a point were used in the extraction calculation, while raster cells whose centroids did not fall within the buffer are excluded^41^. The EVI was calculated by measuring the density of green leaves in the near-infrared and visible bands. The average EVI was calculated for the cells whose centroid was within the radius of 2 km^41^. In addition to other vegetation indices, the EVI has frequently been used in studies assessing greenness and health relationships^42–46^. EVI value ranged between 0 (least vegetation) and 10,000 (most vegetation)^41,47^, and rescaled them to conventional vegetation index values (0–1)^48^. It should be noted that geolocated data were randomly displaced 0–2 km in urban areas to protect the confidentiality of respondents^47^. As a result, a large buffer (e.g., 2 km radius) was used to extract EVI values in order to minimise the effects of cluster displacement.

#### Covariates and mediators

A set of variables was considered in this study based on earlier literature to adjust for confounding^21^, including age (years); sex (male, female); and education status (no or preschool, primary, secondary, college, or higher), working status (working, not working), and marital status (currently married, others [divorced/separated/deserted/widowed/never married]). Based on earlier studies, BMI (kg/m^2^) was selected as candidate mediator^21^.

### Statistical analysis

Descriptive analyses were carried out to explore the distribution of the analytic sample. In addition, the bar plot was used to present the distribution of diabetes across different sociodemographic groups. In this study, a binary logistic regression model was fitted to estimate associations between greenness and diabetes. The greenness variable (i.e., EVI) was standardised before including in the models. The results of regression models were presented as odds ratios (ORs) and 95% confidence intervals (CIs) as well as p values. All analyses were adjusted for the complex survey design and sampling weights. This study used two levels of covariate adjustments: crude models (unadjusted) and main models (adjusted for age, sex, educational status, working status, and marital status). Pseudo R^2^ and Akaike information criteria (AIC) were used to compare unadjusted and adjusted models, with a greater R^2^ and lower AIC values indicating that the model better predicts the diabetes.

This study performed a set of sensitivity analyses. Associations between greenness and diabetes might vary in population subgroups; therefore, this study performed the stratified analysis by age (≤ average vs > average), sex, education status, working status, and marital status as part of sensitivity analysis. The significance of the effect modification was estimated using an interaction term in the logistic regression models. An additional analysis was performed using interquartile range-based categorisation of EVI (low: ≤ 25th, medium: 25th—75th, and high: > 75th) in relation to diabetes.

A mediation analysis was carried out to assess whether BMI was considered a mechanism through which greenness affected diabetes. Standard errors were generated by bootstrapping (5000 simulations), and an *α* level of 0.05 was used for statistical significance. All analyses were performed in *R* (version 3.6.3)^49^.

## Results

### Descriptive statistics

**Figure 1:**
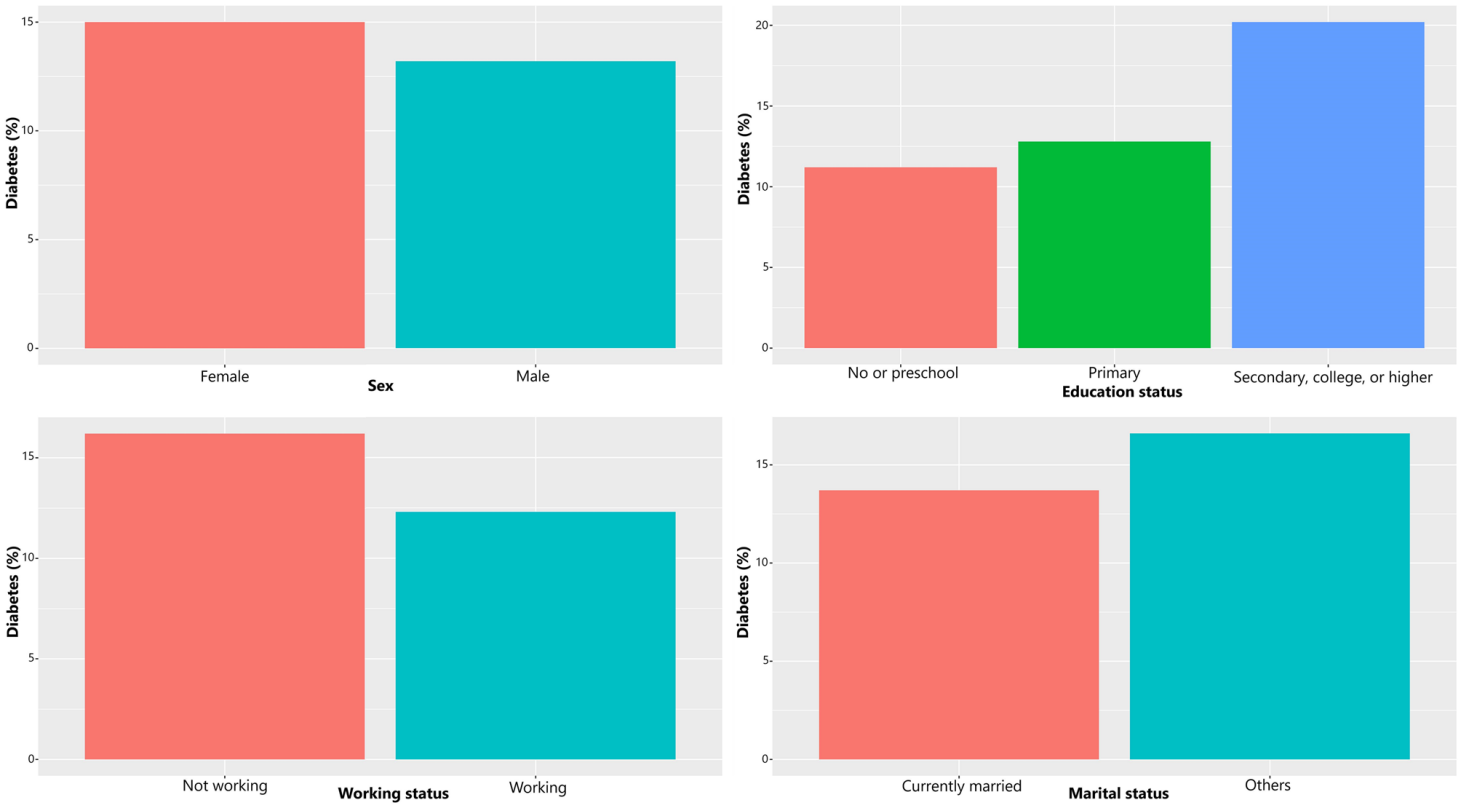
Distribution of diabetes by selected sociodemographic factors.

The prevalence of diabetes among urban adults was 14.1% (Table 1). The respondents’ average age was 49.3 years, and the male to female ratio was 51.8: 48.2. The majority of individuals (87.2%) were currently married, 53.9% had no or preschool education, and 53.7% were working. The average BMI of respondents was about 22.6 kg/m^2^. Among 205 clusters, the average EVI was 0.2657 (SD 0.6832). Moreover, diabetes prevalence was notably greater among female, educated, currently not working individuals and individuals with marital status ‘others’ (Fig. 1).

**Table 1:** Descriptive statistics of the analytic sample.

Variables	Weighted estimate (%)
**Diabetes prevalence**	14.1
**Age** (average ± SD)	49.3 ± 11.6
**Sex**	
Female	48.2
Male	51.8
**Education status**	
No or preschool	53.9
Primary	17.0
Secondary, college, or higher	29.1
**Working status**	
Working	53.7
Not working	46.2
**Marital status**	
Currently married	87.2
Others	12.8
**BMI** (average ± SD)	22.6 ± 4.2
**Cluster level characteristics**	
**EVI** (average ± SD)	0.2657 ± 0.6832 Range 0.0137–0.3847

*SD* standard deviation, *BMI* body mass index, *EVI* enhanced vegetation index.

### Inferential results

Greater area greenness was inversely associated with diabetes among urban adults (adjusted OR 0.805, 95% CI 0.693–0.935, p = 0.0052) after adjusting for covariates. That means 1 SD increase in EVI was significantly associated with 19.5% lower odds of having diabetes. The odds ratio did not change notably in the adjusted model compared to the unadjusted model (Table 2). However, according to Pseudo R^2^ and AIC, the adjusted model was performed better than the unadjusted model in predicting diabetes.

**Table 2:** Association between residential area greenness (1 SD increase in EVI) and diabetes among adults in Bangladesh.

Model specification	Model 1		Model 2	
Predictors	COR (95% CI)	p value	AOR (95% CI)	p value
EVI (standardised)	0.806 (0.694–0.936)	0.0051	0.805 (0.693–0.935)	0.0052
Pseudo R^2^	0.0062		0.0365	
AIC	1914.157		1855.840	

*COR* crude odds ratio, *AOR* adjusted odds ratio, *CI* confidence interval, *EVI* enhanced vegetation index, *AIC* Akaike information criteria; Model 2 is adjusted for individual-level age, sex, education status, working status, and marital status.

#### Stratified analysis and checking effect modification

Stratified analysis (Table 3) by age, sex, educational, working, and marital status revealed that there was a significant inverse association between greenness and diabetes among all age groups, female, adults with no or preschool education, currently not working, and all marital status groups. Greater greenness was associated with lower odds of diabetes among adults aged ≤ average (i.e., 49.3 years) and > average. For females, greater area greenness was also associated with lower odds of diabetes, which was not significant for males. Also, greater greenness was related to lower odds of diabetes among no or preschool educated adults. Living in areas with greater greenness was associated with lower odds of diabetes in adults who were not working and were currently married or others. However, this study did not observe any effect modification by age, sex, educational status, working status, and marital status as the interaction effects were not statistically significant.

**Table 3:** Associations between residential area greenness (1 SD increase in EVI) and diabetes, stratified by sociodemographic variables.

Predictors	AOR (95% CI)	p value	p value for effect modification
**Age**^**a**^			
≤ 49.3 years (average)	0.811 (0.674–0.976)	0.0278	Non-significant
> 49.3 years (average)	0.799 (0.653–0.977)	0.0304	
**Sex**^**b**^			
Male	0.886 (0.714–1.099)	0.2712	Non-significant
Female	0.730 (0.607–0.877)	0.0010	
**Education status**^**c**^			
No or preschool	0.730 (0.586–0.909)	0.0055	Non-significant
Primary	1.000 (0.722–1.386)	0.9978	
Secondary, college, or higher	0.810 (0.635–1.032)	0.0899	
**Working status**^**d**^			
Working	0.823 (0.663–1.023)	0.0805	Non-significant
Not working	0.792 (0.659–0.953)	0.0145	
**Marital status**^**e**^			
Currently married	0.834 (0.707–0.983)	0.0314	Non-significant
Others	0.646 (0.483–0.864)	0.0039	

*AOR* adjusted odds ratio, *CI* confidence interval.

^a^Adjusted for individual-level sex, education status, working status, and marital status; ^b^Adjusted for individual-level age, education status, working status, and marital status; ^c^Adjusted for individual-level age, sex, working status, and marital status; ^d^Adjusted for individual-level age, sex, education status, and marital status; ^e^Adjusted for individual-level age, sex, education status, and working status.

#### EVI categorisation

Adults resided in medium and high greenness urban areas (according to EVI interquartile range) had lower odds of having diabetes, compared to adults resided in low greenness urban areas (Table 4). This result also supports our main results in Table 1.

**Table 4:** Association between residential area greenness (EVI category) and diabetes among adults in Bangladesh.

Predictors	AOR (95% CI)	p value
**EVI category (reference: Low)**		
Medium	0.677 (0.483–0.948)	0.0242
High	0.417 (0.267–0.652)	0.0002
Pseudo R^2^	0.0409	
AIC	1847.626	

*AOR* adjusted odds ratio, *CI* confidence interval, *AIC* Akaike information criteria; model is adjusted for individual-level age, sex, education status, working status, and marital status.

### Mediation analysis

Mediation analysis shows that BMI partly mediated the greenness-diabetes association, explaining about 36.4% of the association, which was statistically significant (p < 0.001) (Table 5).

**Table 5:** Proportion mediated by body mass index in the association of greenness with diabetes.

Mediator	Proportion mediated (95% CI)	p value
BMI	0.3635 (0.2029–0.6200)	< 0.001

*BMI* body mass index, *CI* confidence interval.

## Discussion

The study outcomes extracted from the 2367 Bangladeshi urban adults highlighted the inverse association between greenness and diabetes prevalence, as higher greenness results in lower diabetes occurrence. Stratified analysis showed a difference in the association between greenness and diabetes across sociodemographic groups; however, there was no significant effect modification (interaction effect) noticed. Categorisation of greenness indicator (i.e., EVI) revealed adults living in medium and high greenness areas were less likely to have diabetes relative to adults living in low greenness areas. Mediation analysis shows that BMI partially mediated the association. This is a novel finding in Bangladesh’s context, which inferred the need for an urban natural environment.

Previous studies on the relationship of diabetes with built-environmental features observed that exposure to green space impacts diabetes prevalence. The inverse relationship between greener neighbourhoods and diabetes have already reported by large cross-sectional studies, including 345,143 samples in the Netherlands^50^, 267,072 in Australia (≥ 45 years)^17^, 249,405 in the USA (≥ 65 years)^18^, 3,920,000 in Canada^19^, and 15,477 in China^21^. Longitudinal studies have also found a similar association between greener space and diabetes^20,51^. Nevertheless, few studies also offered results of no association between them^52,53^. However, to contribute to the literature gap regarding similar research on developing countries, the current study reaches a similar conclusion for Bangladesh in line with existing literature.

The stratified analysis of this study professed a link between greener residential areas and cases of diabetes among all age groups analysed in this study. The residential greenness relationship with reduced diabetes has been observed in ≤ 49.3 and > 49.3 age groups, consistent with the previous findings^21^. Results also revealed that higher greenness was strongly associated with lower odds of diabetes among females, no or preschool educated, and non-working adults only. The protective relationship between greenness and diabetes among females observed in this study is also evident from the earlier study in China in which greater greenness (NDVI_1000 m_) was both associated with a 10% reduction in the odds of diabetes^22^. Evidence of an inverse association between greenness and diabetes among no or preschool educated adults is partially supported by findings from an earlier study in which greater greenness was associated with lesser odds of diabetes among adults with lesser education (< 9 years)^21^. The findings of the inverse associations between residential greenness and diabetes were also observed among currently married and other adults, supported by previous research^46^. Although stratified analyses show a significant association between greenness and diabetes among adults in various sociodemographic groups, there is no significant effect modification (interaction effect) by sociodemographic variables, which supports previous literature.

Despite the lack of effect modification, the stratified analysis results can help to generate new hypotheses, and invite future research. While few sociodemographic groups were more hampered by residential area greenness than others, as the modification effect was non-significant, they are outside the scope of this study. However, more data and in-depth understanding of each vulnerable cohort is required to explain these results and validate these findings.

Recent research has also shown that higher greenness is related to a reduced risk of obesity^54–57^, an important risk factor for diabetes^58^. This study, therefore, assessed the mediation effect of BMI on the relationship between greenness and diabetes in the residential area and found a significant effect, which is supported by an earlier study^21^. BMI explained about 36.4% of the association between greenness and diabetes among adults. Thus, greenness could play an important role in diabetes prevention by decrementing obesity. Possible explanations include air pollutions increases and average physical activity decreases as green spaces disappear from the neighbourhood, which is associated with obesity^31,59^. However, future studies could assess seating time, rate of physical activity and dietary behaviour to focus on how these factors mediate the association between environment and diabetes prevalence.

Several mechanisms could explain the inverse relationship between greenness and diabetes, such as improved air quality, increased physical activity and less sedentary behaviour. Air pollution can be related to diabetes by various biological pathways such as endothelial dysfunction, dysregulation of the visceral adipose tissue, and hepatic insulin resistance^60^; however, greenness is expected to reduce air pollution, increase the supply of oxygen and decrease the risk of diabetes. Similarly, neighbourhoods with playgrounds, parks, and gardens are found to encourage, facilitate and reinforce physical activity^61^ and discourage sedentary behaviour, resulting in reduced diabetes risk. Thus, if Bangladesh intends to fight the increasing prevalence of diabetes, it must design the metropolitan cities such as Dhaka and Chittagong following leading examples of Vienna, Sydney, or Melbourne.

The current study has some limitations. First, cross-sectional design restricted from making any causal link between greenness and risk of diabetes; therefore, reverse causality cannot be ruled out. Second, individuals’ exact geocoded residential addresses were not provided in the survey data used in this study but rather cluster (or community) centroids. These area centroids were also used to extract environmental features (i.e., EVI). As a result, they may create situations that result in misclassification of exposure. However, previous studies have used cluster or community centroid-based greenness exposure^21,57,62^. In addition, the survey authority displaced the survey cluster locations at random for confidentiality purposes, which may have contributed to the exposure misclassification error. Third, this survey lacked information on time spent in residential areas and green space near the workplace, which could lead to bias and residual confounding. Fourth, this study was unable to determine the impact of different types of greenspaces on diabetes since EVI does not include data on the quality and structure of greenspaces. Fifth, data were collected in 2011, and no national survey in Bangladesh collected similar information on diabetes and vegetation index. Thus, this study was limited to slightly older data; however, the findings emphasise the need for DHS to collect more biomarker measurements and vegetation data in future surveys. Sixth, this survey used EVI to generate vegetation values in 2 km buffers around the cluster centroid, which limited the scope to run any sensitivity analysis of observed relationships for specific buffers (e.g., 0.5 km, 1 km) or assess using other vegetation measures (e.g., NDVI). Future data collection efforts could include multiple vegetation indices for greater specificity and sensitivity with smaller buffer areas. Finally, data shortage on physical activity, food habits, and environmental components could be possible risk factors for blood sugar levels and were not adjusted in the study models. Future data collection efforts may include these for more robust findings. Given these limitations, the findings of this investigation should be interpreted with caution and validated in future prospective studies. The generalisation of results from developed countries to developing countries is often challenging, owing to different definitions of greenspace or greenness^33,63^. Thus, the current study could be a basis to assess the association between urban greenness and NCDs in Bangladesh.

## Conclusions

The antithetical relation of residential greenness and diabetes prevalence meaningfully describes the benefits of higher residential greenness in metropolitan cities of Bangladesh. Furthermore, as hypothesised, the association was partially mediated by BMI. The findings are novel in the context of Bangladesh that will authenticate as advantageous for public health experts, urban planners and policymakers to review diabetes prevention policies and emphasise the importance of residential area built-environmental features to mitigate the rise in diabetes among urban Bangladesh’s population. More studies, preferably longitudinal, are needed to continuously monitor the environmental changes and NCD prevalence in Bangladesh to draw a causal conclusion with policy directives.
